# Health and Risk Behaviors of Bystanders: An Integrative Theoretical Model of Bystanders’ Reactions to Mistreatment

**DOI:** 10.3390/ijerph18115552

**Published:** 2021-05-22

**Authors:** Yariv Itzkovich, Ella Barhon, Rachel Lev-Wiesel

**Affiliations:** 1 Kinneret College on the Sea of Galilee, Tzemach 1410502, Israel; ea.barhon@mx.kinneret.ac.il; 2 School for Creative Arts Therapies, University of Haifa, Haifa 3498838, Israel; rlev@univ.haifa.ac.il

**Keywords:** theoretical model, bystanders’ responses, mistreatment, adolescents’ risk and health risk behaviors, conservation of resources theory, potency, moral disengagement

## Abstract

This article constructs a comprehensive theoretical model that outlines bystanders’ emotional and behavioral responses to the mistreatment of adolescent peers. The model captures bystanders’ risk and health risk behaviors, which have been overlooked in the context of their reactions; when addressed at all in connection with bystanders of bullying among adolescents, they have been treated separately. Here, we present bystanders’ emotional and cognitive reactions and their impact on bystanders’ responses including a set of responses that demonstrate risk and health risk behaviors that are directed to the bystander as a victim by proxy. The theoretical framework is the conservation of resources theory, which posits that personal resources (i.e., potency and moral disengagement) and social resources impact the process that leads to bystanders’ reactions. Previous models have overlooked the integrative viewpoint of bystanders, and comprehensive models that explain bystanders’ behavioral and emotional responses have received little attention especially with regards to adolescents. Two recent models overlooked core features embedded in the current model, including the risk and health risk behaviors that it integrates. The proposed model presents a novel and more comprehensive view of bystanders’ reactions and the process underlying these reactions. It integrates existing knowledge embedded in other existing models. At the same time, this perspective indicates the centricity of potency as a key resource that dictates the emotional response and behaviors of bystanders. This potentially allows for new applications in the mitigation of adverse impacts that follow the witnessing of mistreatment. The article discusses these applications, which are based on previous findings, their implications for practice, and directions for future empirical research necessary to validate the model.

## 1. Introduction

Bullying among adolescents in schools has been widely addressed [1]. The plethora of research on the subject [2], however, has focused mainly on the dyadic interplay between bullies and victims [3,4]. As bullying rarely occurs without others observing it, a comprehensive viewpoint for the study of bullying should include bystanders, who are the largest group impacted by bullying, by either directly observing acts of bullying or by being exposed to bullying mediated by technology [5,6,7].

One approach to the study of bystanders of bullying considers bystanders as victims by proxy. This line of research has focused on how witnessing bullying acts impacts the well-being and psychological health of bystanders [7]. It has demonstrated a correlation between witnessing an act of bullying and suicide ideation [8], symptoms of depression among bystanders [9,10,11], especially among those who were exposed to bullying [12], repression of empathy of bystanders [13], and increased feelings of guilt [4]. The underlying assumption of this research approach is that, in the act of bullying, bystanders are passively victimized. Thus, although bystanders are not part of the dyadic conflict between the perpetrator and the victim, they are passively exposed to mistreatment through their observation in a way that may damage their sense of self. To date, no existing model describes the triggers of bystanders’ emotional and behavioral responses, as victims by proxy.

The antecedents of bystanders’ victimization have been scantly addressed [14]. Interestingly, it has been highlighted that victims and bystanders share some antecedents for victimization, whether directly as targets or indirectly as bystanders. Indirect victimization was found to be associated with social resources, such as household socioeconomic status and parental efficacy. It is also associated with personal attributes, such as self-perceived ability to avoid violence and former experiences with bullying [15]. However, there is not yet any theoretical model suggesting a framework for understanding the process of bystanders’ victimization.

Other researchers have adopted a wider perspective, as noted by [16], noting that bystanders are not merely victims by proxy. The underlying assumption of this approach is that bystanders’ behaviors and actions can have pronounced effects on all elements of the bullying process and, more specifically, on the continuation or inhibition of bullying [17]. These impacts consist of various emotional and behavioral responses of the bystander, some of which are constructive (either active or passive), driven by the bystander’s willingness to help the victim because of a sense of responsibility [7,17].

Other reactions are destructive [7,18]. Although active-destructive behaviors are driven by a belief that the victim deserves to be mistreated and thus bystanders actively become part of the perpetration, passive-destructive behaviors are avoidance reactions that enhance the offender’s sense of control, power, and position. In this sense, especially when adolescents shaping their identity are involved, the audience (i.e., passive bystanders) are needed and used by the perpetrator, and, as such, they also shape their own identity as a mere audience (the sheep role).

Apart from a small number of attempts to adopt a broader perspective on the roles of bystanders, such as the study by Chen et al. [18], which mentions all six roles of bystanders (including as victims), these two viewpoints namely ‘victim by proxy’ and ‘bystander as part of the victimization process’ have for the most part been addressed separately. Moreover, the focus on bystanders as victims, beyond the mental implications noted, has resulted in a neglect of the behavioral responses of witnesses directed toward himself or herself [19,20]. Conceptually, looking at risk and health risk behaviors as bystanders’ reactions to bullying can bridge the gap between the two separate perspectives mentioned above. It allows the integration of the view of bystanders as victims by proxy with the alternative view that bystanders are part of the process under a unified set of behaviors.

Looking at the complete portfolio of reactions raises two profound questions concerning the determinants of the different perspectives and the process that directs them. The first question focuses on the nature of the factors that determine the bystander’s choice, and the second concerns the process underlying this choice.

In response to the first question, scholars have focused on different determinants, although none have provided a complete model that addresses multiple antecedents. In this regard, Gaete et al. focused on former experience as an antecedent for substance abuse among bystanders [5]; Hutchinson focused on the social context of bystanders and the psychological costs of bystanding [4]; Knauf et al. focused on various determinants such as moral disengagement, empathy and self-efficacy, and feelings of responsibility as antecedents of bystanders’ reactions [6]; and Espelage et al. focused on age, gender, social context (i.e., norms), willingness to intervene, and attitudes toward bullying [17]. There remains a need to adopt a more comprehensive viewpoint that takes full account of these antecedents and more importantly, their underlying triggers.

The second question concerns the process that directs the different perspectives. Thus far, various studies have adopted the model proposed by Latané and Darley [21], which sets out a five-step orbit for bystander intervention: (1) noticing an event, (2) recognizing the need for action, (3) taking personal responsibility, (4) choosing an intervention, and (5) implementing the intervention. This model has been utilized in social abuse situations, namely bullying [6], and it was recently applied to bystanders’ roles [22]. Nonetheless, as noted by Knauf et al. [6], there remains a need for a profound understanding of the affective and cognitive process underlying bystanders’ decisions.

In this respect, two models have been proposed concerning bystanders’ reactions to workplace bullying that have the potential to address this gap and that integrate different responses into a single model [23,24]. These models provide a more integrative view of bystanders’ reactions by seeing them in terms of active and passive or constructive and destructive responses based on the work of Paull et al. 2012 [16]. In a recent study [24], Niven et al. answered Knauf et al.’s call [6], outlining a cognitive-emotional process triggered by witnessing acts of bullying and igniting a set of active–passive–constructive–destructive responses driven by emotions. Although this illuminating approach captures a broader range of reactions, it has three lacunas. First, the authors overlook the dynamic nature of emotions as a trigger to a dynamic set of responses, as described by Dolev et al. [25]. Second, they neglect the implications of these reactions for future events beyond the repeated bully–perpetrator interaction, including hypervigilance of the bystander in future unrelated events. Lastly, their model ignores the behaviors of the bystander that affect the bystander himself or herself, namely risk and health risk behaviors. Ng et al. presented a different model based on this approach. The authors proposed a dynamic model that considers the transformation of behaviors over time in a continuous bullying episode [23]. Their groundbreaking model embedded Bandura et al.’s conceptualization of moral disengagement [26], as suggested by Knauf et al. [6]. However, it fails to capture behaviors directed toward the self, namely the risk and health risk behaviors of bystanders [20,27], overlooking the role of emotions in the ongoing process and the impact on bystanders’ future hypervigilance in future distinct episodes of bullying.

Thus, to address these gaps, the present article presents a wider view of bystanders’ reactions and the underlying process, with the goal of providing a comprehensive model that includes risk and health risk behaviors as representations of the victims-by-proxy approach.

The proposed model will also illustrate an ongoing process that follows bystanders’ reactions in a continuous circular process. Unlike its predecessors, the model considers the dynamic nature of emotions, and the dynamic nature of behavior over time, respective to changes in individuals and occurrences.

Although classic models completely overlook the recurrent nature of bullying [28] current enlightening models [23,24] account for the dynamic nature of witnessing an act of bullying, and its implications. However, even they overlook a broader perspective that extends beyond the dyadic or triadic equation of a particular recurrent act. The proposed model is in line with findings demonstrating that the passive experience of bystanders’ victimization increases their likelihood for future observation of unrelated incidents. This finding is also supported by other theoretic models that explain violation of psychological contracts [29]. The work of Salin and Notelaers [30], for example, shows that being a bystander to bullying can be seen as a violation of a psychological contract.

Additionally, these models view of emotions is based on the notion that specific emotions elicit specific behaviors [24], yet our view is more dynamic in line with recent proposed dynamic view of emotions [25].

The current model integrates knowledge embedded in existing models, allowing a wider view of bystanders’ motivations and behaviors. It also indicates the centricity of potency and social resources as key resources that dictate the emotional responses and behaviors of bystanders. This allows for novel applications in mitigating the adverse impacts after witnessing mistreatment, especially among adolescents, as informed by similar contexts. Previous findings of studies among adolescents-at-risk indicate that strengthening potency, especially the two factors of belief in a just society and social support, serve as a buffer against drug abuse [31]. No previous model describes the triggers of the emotional and behavioral responses of bystanders as victims by proxy. By doing so, the proposed model addresses the notion raised in Paull et al. [16], emphasizing that effective prevention and intervention strategies should recognize bystanders’ multiple roles.

Additionally, other existing models focus on explaining the phenomenon of witnessing bullying in work settings. In contrast, the current model is designed to explain bystanders who are adolescents. Despite their commonalities, it seems that being a bystander elicits higher levels of distress and greater emotional impact on adolescents than on adult employees [16]. Thus, a separate model is needed to account for the process and its implications among adolescents. Moreover, other models utilize various antecedents to explain bystanders’ reactions, some of which are explanatory variables focusing on situational traits, such as the time course of the act. Thus, despite their explanatory contribution, they contribute less to mitigation. Using the conservation of resources (COR) theory as a framework to explain bystanders’ reactions to bullying can help mitigate adverse impacts, by enhancing the resources that are key features in the current model.

In summary, looking at these processes from a COR perspective enables the development of a dynamic view of bystanding, present and future implications beyond recurrent triadic interplay, a comprehensive view of the phenomenon, and directions for mitigation of risk and health risk behaviors.

## 2. The Framework of the Proposed Model

The conservation of resources (COR) theory, used here as a theoretical framework, proposes a dynamic model of stress that helps us to understand how individuals’ coping resources function in the process of reducing their exposure to stressors [32,33,34,35]. Studies have consistently shown that individual psychological differences lead to the adoption of different coping strategies and other emotional and regulatory resources in the face of difficult situations [25]. In 30 years of research, COR has been used in a wide array of stress-related situations, mostly in organizations [33], but also to explain social rejection among adolescents [36].

The underlying assumptions of COR make it appropriate for understanding the drivers and underlying process of bystanders’ reactions based on individual responses to a complicated sequence of stressful conditions that occur over time [33]. In that sense, it takes into account the dynamicity of stress and the process underlying it.

Thus far, previous models have used frameworks that highlight various facets of bystanding, either centered on the cognitive facet [23], on cognitive and emotional facets [24] or merely on the typology of bystanders’ reactions [16] as summarized in Table 1.

COR is utilized as the framework of the current model for two main reasons. First, it focuses on an ongoing dynamic process that accounts for the impact of current resources and coping on future resources and coping beyond a specific event. Second, since it focuses on resources, it points to possible interventions. Thus, if resources or lack of resources dictate behavior, cultivating deficient resources will allow for future extinction of adverse behaviors once these scarcities are addressed, as shown by previous findings in similar contexts [31].

COR theory is based on four underlying assumptions. First, it recognizes that people are motivated by resource loss more than they are motivated by resource gain. Second, it postulates that people must invest resources to protect against resource loss, recover from loss, or gain resources. Third, it emphasizes that resource gain is more prominent in the context of resource loss. Fourth, it notes that when their resources are overstretched or exhausted, individuals enter a defensive mode to preserve the self, and that this is often defensive or aggressive in form, and may become irrational [33]. Moreover, the authors stress that, over time, loss of resources impacts the level of resources in hand that could be used in future stressful events, thus illustrating both the dynamicity of processes and their predictive power.

Although COR was initially used in organizational settings, it has been embraced by scholars to explain social rejection among adolescents [36]. In this respect, potency (a personal resource) and social support (a social resource) have been considered as resources that buffer the interrelations between social rejection, depression, and post-traumatic stress drivers. In the framework of the current model, these resources will explain bystanders’ cognitive, emotional, and behavioral reactions.

The ability of individuals to achieve specific goals is conditioned by their personal resources, which are defined as traits that enable them to deal with adverse life events and stressful situations [40,41,42]. These traits include potency [43], which is defined as self-control, self-confidence, and as trusting in society and social support. Unlike self-efficacy, self-esteem, and resilience, which refer mainly to a person’s intrapersonal resources and are manifested through a sense of mastery, the concept of potency beyond its self-centered focus concerns the individual’s commitment to a social environment that is perceived as basically meaningful, predictable, and moral [41]. In addition, the concept of moral disengagement explains risk and health risk behaviors as part of the model.

Moral disengagement (MD) theory focuses on the processes by which self-regulatory mechanisms are deactivated to maintain a moral image of oneself, eliciting unethical behaviors without violating internal standards of morality [44], and without producing feelings of remorse, guilt, or shame [45]. As ethical and unethical behaviors are products of the reciprocal interplay between personal and social influences and are, thus, socially embedded [44], it is to be expected that once MD is activated it will be socially learned by others. Bandura has argued that the relationship between moral reasoning and action is mediated by MD, a self-regulatory process that enables moral agency and helps individuals to reduce tensions associated with unethical behaviors [44]. In particular, Bandura suggested eight mechanisms that enhance MD by distortion of moral judgment: moral justification, euphemistic language, advantageous comparison, distortion of consequences, diffusion of responsibility, displacement of responsibility, attribution of blame, and dehumanization [44]. We suggest that bystanders may use some of these mechanisms to justify their reactions toward the victim and perpetrator, and their self-risk and health risk behaviors in connection with their inventory of resources. Indeed, former studies identified MD as a rationalization mechanism that is used by bystanders to explain their pro-aggressive behavior [46,47] or their inaction [48]. Utilizing COR as a conceptual framework sheds light on the underlying logic of using MD as specified as part of the presentation of the model.

**Figure 1:**
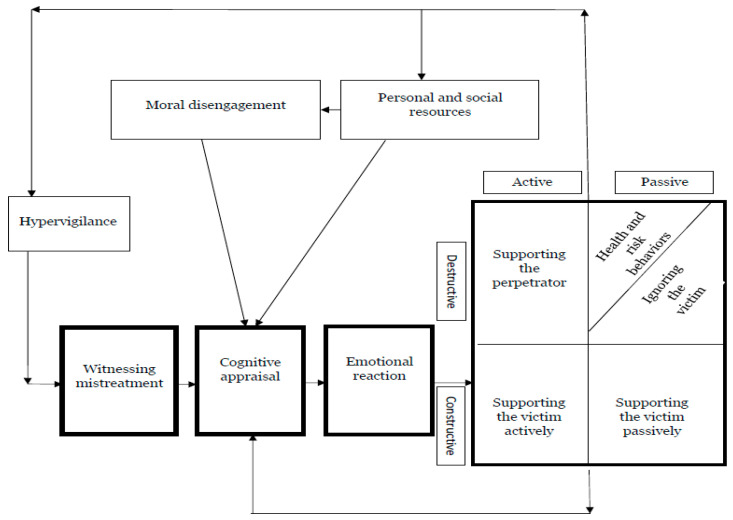
Process and dynamics of bystanders’ reactions in the framework of COR: the proposed model.

Figure 1 provides an overview of our model, which starts with the suggestion that the observation of bullying triggers a process leading to bystanders’ responses. Once bullying is observed, a cognitive appraisal process is triggered [23,24], followed by an emotional response [24]. Emotions provide invaluable self-information and information about various interactions between individuals and their environments [49], and the cognitive appraisals underlying emotions and emotional responses are crucial to the study of emotional experiences [50]. According to Lazarus’s theory of the cognitive appraisal of emotions [42], cognitive appraisal is a process by which individuals assess why and to what extent social encounters are stressful. At the same time, coping is the processes by which individuals manage the demands of person–environment relationships and their emotions [50]. According to Lazarus and Folkman [42], psychological stress occurs when individuals appraise relationships with their environments as potentially damaging to their well-being. In particular, it has been argued that negative appraisals of an experience (i.e., observing an act of bullying) induce negative emotions that trigger bystander reactions.

In this respect, active emotions such as anger, which are based on high levels of personal resources, have been found to lead to actions aimed at supporting the victim, while passive emotions such as fear can lead to avoidance [25]. Other scholars have emphasized that fear can lead to withdrawal behavior, and that anger can lead to active support for the victim. Niven et al. [24] also noted that schadenfreude may lead to the re-victimization of the victim and that sympathy may lead to passively helping the victim. Passive and active emotions may coexist as part of a single reaction and change over time [25]. Thus, based on Ng et al. [23], we can view emotions in a way that recognizes appraisal as an ongoing dynamic process.

The existing models, as described in Table 1, explain the process, its cognitive appraisal, and the emotional response elicited. Although they account for antecedents of the cognitive appraisal [23,24], some of the antecedents they account for, such as the timeline of the bullying, cannot be modified, and, thus, have limited contribution as potential mitigators. Other antecedents such as moral values and relative power of the bystander are reflected through potency. Potency is composed of high self-confidence, a heightened sense of control (i.e., relative power), and belief in the existence of a just and supportive society (i.e., moral values). These resources can be impacted, and indeed have been utilized to reduce risk and health risk behaviors [31].

Thus, the COR framework can contribute to the theory of the cognitive appraisal of emotions in three ways [50]. First, it can deepen understanding of the process that underlies the decision concerning a coping strategy. Second, it enables the prediction of future behavior based on current perceived stress and correspondence with future implications for the individual’s resource inventory [33]. Third, since these resources are dynamic and can be obtained or developed, utilizing COR can point to the application of the model in mitigating adverse reactions among bystanders’.

In terms of COR, a cognitive appraisal is focused on both the current threat to one’s resources and the implications that any reaction has for these resources [32,33]. On the one hand, witnessing the act of bullying itself threatens two components of potency, namely the personal perception of self-control and the belief in a just and ordered society [36]. Thus, it calls for action to defend these resources. On the other hand, any future reaction by the bystander may have implications for these and other components of potency, such as individual self-confidence and the individual’s perception of his or her relationship with society. In this sense, we posit that four types of responses can be elicited from the cognitive evaluation and emotional stimuli following it, all of which depend on the inventory of personal and social resources, namely potency and social support. In line with the work of Paull et al. [25], these reactions can be divided into four categories of responses on two dimensions: active–passive and constructive–destructive.

Individuals with high potency (i.e., high self-confidence, a heightened sense of control, and belief in the existence of a just and supportive society) will be motivated and cognitively tuned to supporting the victim actively. Such support is shaped by their potency [41], will help them to maintain their future potency, especially in relation to their view of society, and will presumably restore peace, plausibly identified as a resource [32]. In this regard, especially if individuals have social support, they can actively confront the perpetrator or call for external assistance [17]. This notion leans on the social setting and personal resources nourished from the social environment [32]. Previous research has identified various antecedents of active support toward the victim, including empathy [51], willingness to intervene [17], gender (which is considered to be an antecedent of empathy) [17,51], and taking responsibility [2], all of which can be regarded as embedded within potency.

When individuals cognitively evaluate that active confrontation with the perpetrator will jeopardize some of their resources, they can still support the victim passively [23]. In such cases, they can maintain their potency, regarding their commitment to society and their belief in a just world, without jeopardizing other potency components, such as their self-confidence, that might be required and challenged when confronting a strong perpetrator.

The two other types of reactions suggested in our model can be categorized as destructive. First, bystanders can actively support the bully in a set of responses identified in the literature as reinforcers [2]. We posit that such behavior is more prevalent among individuals with low potency, who do not believe in a just world or in an orderly, just society [36], as well as in their own ability to make the world just. Additionally, that these individuals have a low inventory of social resources, such as family and neighborhood social resources [14,15], becomes a risk factor for engaging in perpetration of bullying [36].

However, we believe that the understanding of MD above and beyond accounting for low resources (i.e., potency and social resources) are needed to explain the willingness to help the perpetrator and to overlook the feelings and overall experience of the victim.

We stress that individuals who ignore an act of bullying are likely to develop feelings of guilt and remorse. These will impact their future self-esteem resources, which are part of their potency [49]. This notion relies on the work of Hutchinson [4], who found that bystanders’ feelings of guilt about their inaction challenge their self-esteem. Alternatively, actively helping the perpetrator may be rationalized and normalized by bystanders who sympathize with the perpetrator [52] to defend their self-perception as moral individuals and to protect their social resources in terms of their place in their community, as informed by socio-ecological theory [53]. Thus these individuals may use MD as a defensive shield, although for different reasons. Indeed, findings from various studies indicate a connection between moral disengagement and destructive reactions of bystanders, whether passive or active, as explained through MD [54]. Although these findings help us to understand the interrelations among MD and bystander reactions, they are not grounded in a comprehensive theoretical framework that accounts for the interrelations between individual resources and MD as predictors of bystander’s appraisal, emotional response, and reactions.

The difference between active– and passive–destructive behavior may depend on the use of different mechanisms of disengagement. Attribution of fault to the victim (“Some kids get bullied because they deserve it”) or cognitive restructuring (“It’s okay to join in when someone you don’t like is being bullied”) can allow the bystander to cooperate with the bully. Avoiding the victim may depend on a distortion of the negative consequences (“Getting bullied helps to make people tougher”) or on a minimization of agency (“Adults at school should be responsible for protecting kids from bullies”) [54] (p. 5).

Using COR and MD allows us to explore a further set of passive–destructive bystander behaviors, namely risk and health risk behaviors. Incorporating risk behaviors into a unified model of bystanders’ reactions makes it possible to encompass two complementary viewpoints that have so far been addressed only separately, namely the bystander as a victim by proxy, and the bystander as a player in the act of bullying and a part of its process.

## 3. Health and Risk Behaviors of Bystanders in the Framework of COR and Moral Disengagement

In our proposed model, we suggest another set of bystander responses to bullying that have so far been overlooked. These reactions can be categorized as passive–destructive, although they are in some respects distinct from other responses in that category. Unlike the other passive–destructive behaviors presented here, these behaviors are directed toward the bystander himself or herself.

Studies have found a link between bullying behaviors and substance use among adolescents [55,56,57]. Specifically, findings indicate a strong association between legal substance use and being a victim of bullying [58], which is in line with studies that have identified the use of illegal drugs, such as marijuana, as a reaction to victimization from bullying [59]. Although the line of research focusing on bystanders’ risk and health risk behaviors is scantly addressed [60], some studies found that exposure to violence, either directly as victims or indirectly as bystanders, equally increases internalization of suicidal ideation, substance abuse, and self-directed violent behavior (e.g., attempted suicide) among adolescents [14]. Similarly, scholars found that bystanders and victims to bullying had similar risks for cigarette smoking, alcohol consumption, and cannabis use. [5]

It has been established that victimization triggers a similar emotional and physical impact both on victims and on bystanders of bullying. In particular, repetitive abuse can affect bystanders and victims when the events occur later in life [13]. Thus, it can be assumed that bystanders may also consume substances after exposure to bullying. Indeed, Gaete et al. observed that bystanders used legal and illegal substances following their bullying experience [5], and they concluded that distress and helplessness are rooted in these risk and health risk behaviors. Supporting evidence is found in the interrelation of bullying with suicidal ideation [8].

In the COR framework, although low potency makes these bystanders reluctant to defend victims of bullying, they still have to deal with their helplessness and feelings of sympathy toward the victim. They are morally distressed, as they feel the need to help but lack the ability (or courage) to do so [5]. Despite their empathy for the victim, their lack of social self-efficacy resources serves to elicit feelings of fear and empathy combined [61]. Byers argues that bystanders tend to use MD due to anxiety and frustration as a coping mechanism [61]. Yet, our model indicates that, to cope with the frustration, they may engage in substance use and justify that use in terms of MD in order not to lose more resources. This claim finds support in the work of Basharpoor and Ahmadi, who found MD to be a compelling factor in predicting a tendency toward high-risk behaviors among students [62].

In the framework of COR, we see two additional paths that enable a developmental view of the process. Once risk and health risk behaviors are employed, self-confidence and self-perception are damaged, as Hutchinson implied [4], noting that the inaction is, by itself, enough to trigger the bystander’s shame. In terms of resources, we expect that the chances of such bystanders taking constructive action in recurrent experiences of bullying by standing are reduced, as their resource inventory in terms of their place in society and a sense of worth are reduced, with an impact on subsequent cognitive evaluation that together elicit irrational behaviors when resources are overstretched or exhausted [33]. Specifically, in low potency conditions, individuals feel less capable of helping the bullying victim, and thus their sense of shame triggers risk behaviors that are irrational but directed towards protecting the self.

Furthermore, as COR is an ongoing process, it can also account for future events unrelated to the current bullying incident. Recently, Salin and Notelaers have shown that being a bystander to bullying can be seen as a violation of a psychological contract [30]. Thus, it is reasonable to assume that the process underlying psychological contract violation will explain a bystander’s future reactions. In her illuminating model, Rousseau suggests that, once the contract has been violated, hypervigilance is triggered in the individual whose contract was violated [29]. This, in turn, triggers future bystanding according to the individual’s level of sensitivity to future violence, and, thus, observation of more incidents are to be expected.

## 4. Applications of the Model

Our model and its underlying mechanisms offer directions to scholars and others seeking to intervene and reduce the destructive implications of bystanding, especially on adolescent bystanders. Increasing individuals’ potency and social resources buffers the potential impact of external demands that are confronted by bystanders, such as individual helplessness and social alienation.

Potency is a cluster of the following traits: self-control, self-esteem, belief in personal ability, belief in the existence of a social order and in society as being just and significant [31,36]. Social resources are sources of perceived support from others [31]. Increasing potency and perceived social support were found to reduce risk behaviors among adolescents, [31]. One potential application drawn from Lev-Wiesel’s work [31] is based on the following principles of social learning (as described on page 385), which are primarily designed to enhance potency and perceived social support as resources:

(1) The development of personal attributes, as learned in social situations;
(2) The tendency of youth to use their imaginations to manage their stress;
(3) Physical activity intended to increase self-confidence and self-esteem;
(4) Enhancement of traits that prevent drug abuse and facilitate socialization, such as positive self-image, self-esteem, internal locus of control, and commitment to society;
(5) A rehabilitative and focused treatment program that inculcates skills, problem solving and an anti-criminal model of behavior to bring about a reduction in criminal behavior.

The author developed a workshop focusing on the individual and social resources related to various tasks the participants performed. Her findings revealed that strengthening potency, especially the two factors of belief in a just society and social support, served as a buffer against drug abuse. The last principle in Lev-Wiesel’s application, which is focused on modeling moral behavior and the problem-solving competencies that can enhance it, indicates another potential route of application of the model.

Many intervention programs addressing school violence and bullying are based on skill enhancement and cultivating moral judgments and codes of conduct among students. These are integral parts of values education that have been highlighted as an essential aspect in violence-prevention programs [63].

One of its applications, the values and knowledge education (VaKE) approach, is an intensive training session, which can be employed as a violence prevention strategy. As a learning method, it combines constructivist learning and values education to develop moral judgment competencies [64].

Briefly, during a VaKE learning process, learners face a moral dilemma that raises questions about various solutions. The discussion about the dilemma triggers questions about what is vital to know (i.e., knowledge questions) in order to come up with a solution. In the next step, learners seek answers in relevant sources available.

In a complete two-stage VaKE process, the learners seek: (1) ethical justification in favor or against moral values in the given dilemma situation, and (2) empirical evidence necessary for their argumentation. Thus, they discuss the dilemma in terms of understanding it, build an evidence-based argumentation, and justify it morally [65].

The values involved in the moral dilemma presented to the learners should reflect the participants’ level of moral development to engage them and promote their moral judgments to higher moral levels. The VaKE approach has been implemented in formal and non-formal settings. The literature on VaKE provides support for the argument that VaKE can be used as a prevention strategy against school violence and bullying. Similar to Lev-Weisal’s intervention plan, VaKE emphasizes the development of problem-solving and socio-emotional skills, and, thus, it can also cultivate potency and perceived social resources, key elements in the model [65].

## 5. Discussion

Our model offers an additional perspective on bystander reactions. It accounts for all types of bystander reactions, including those typically not discussed as part of bystanders’ responses, namely risk and health risk behaviors that were scantly addressed. Thus far, no theoretical model accounts for these behaviors. This is the first model to account for these behaviors and to provide a wider perspective that accounts for bystanders’ reactions beyond the triadic interplay and specific recurrent bullying incidents all in the framework of COR.

As a framework, COR allows us to account for various types of responses and the process of bystanding, suggesting a rationale for the different reactions and a developmental viewpoint of the process as a whole. Although Latané and Darley also used COR as a framework for understanding bystander reactions [21], their model had a limited ability to explain the underlying rationale of the various types of responses, and it overlooked the implications of bystanding beyond the current incident. Our proposed model explains the risk and health risk behaviors of bystanders that have received little attention, incorporating them into a model that illustrates the complete range of bystanders’ behaviors. This is also the first time that the two-dimensional typologies of reactions commonly used in workplace bullying research have been used to describe bullying in educational settings [16,23].

Our model also takes account of the dynamic nature of bullying and the dynamic nature of emotions and reactions. Only one previous model has attempted this [24], yet it overlooked risk and health risk behaviors that are one of the main contributions of our model. There has been little exploration of bystanders and health and risk behaviors [5]; the few studies that have addressed the subject lacked an integrative framework.

The COR framework is founded on individuals’ continuous quest for resources. Specifically, the resources outlined in the current model can teach us about potential future measures for prevention, and intervention of bullying acts. Following this model, the enhancement of potency and social resources are key resources. Cultivating these resources can reduce the adverse implications of bullying on bystanders and other parties involved. Although applications of the model for prevention, and intervention should be tested empirically, the model indicates ways to navigate the long road ahead of us toward reducing the costly implications of bullying.

## 6. Future Directions and Research Limitations

It should be noted that while our model provides a broad perspective on bystanding, it is theoretical. Future research should therefore seek to validate its components and their interrelations.

Another direction for future research is utilization of the model in workplace settings. Implications of witnessing bullying, including but not limited to risk and health risk behaviors among employees who are bystanders to bullying, are scantly addressed, although the implications on victims of bullying are widely addressed. In this regard, focusing on risk and health behaviors among employees can take a parallel route, such as the implications on accident rates or risky driving when commuting. Such research has been done, but not on bystanders [65]. This is one of many potential routes of research which can draw on Paull et al. [16], who noted that although “…important differences between school and workplace bullying, these commonalities suggest organizational bystander research can benefit from school studies,” (p. 353).

## 7. Conclusions

Bystanders are the largest population experiencing bullying, although by proxy. As they are highlighted as pivotal players in the mitigation of bullying, it is highly important to understand their underlying dynamics in diverse contexts, to increase the ability to intervene in acts of bullying. Our article offers a broad viewpoint highlighting the dynamic nature of the act and its impact on future unrelated acts, while accounting for an overlooked facet in current models—bystanders’ own victimization.

Focusing on resources (i.e., potency and social resources) allows us to suggest theory-based and evidence-based mitigations of risk and health risk behaviors. For the first time, these have been integrated in a model that explains bystanders’ reactions and motivations.

We call upon future researchers to utilize this model to address the issue of bullying, and especially risk and health risk behaviors of bystanders. We conclude with the hope that applications of our model will increase bystanders’ inclination toward constructive participation in the future, and make them better equipped to oppose bullying.

## Figures and Tables

**Table 1:** Existing models of bystander behavior, following the terminology used by Paull et al. (2012) [16], of passive, active, constructive, and destructive responses.

Source	Purpose	Theoretical Framework	Main Contributions	Shortcomings
Niven et al. (2020) [24]	To describe the cognitive-emotional process triggered by witnessing an act of bullying that ignites a set of active/passive constructive/destructive responses driven by emotions.	Classic theory of action readiness (Frijda, 1986) [37]/Appraisal theory of emotions (Lazarus, 1982) [38]	Identification of the conditions under which particular types of bystander responses emerge. Identification of various personal and situational factors that influence how bullying incidents are appraised and the extent to which emotional reactions determine bystander behavior. The model is focused on workplace bullying.	The authors overlooked the dynamic nature of emotions as a trigger to an active set of responses, as described by Dolev et al. (2020) [25]. The authors overlooked the possible implication of bystanding on future events beyond the bully-perpetrator repeated interaction, namely hypervigilance of the bystander in future unrelated events. The model is centered on some factors such as the time course of bullying and culture that can’t be addressed in mitigation processes. The model does not incorporate risk and health risk behaviors of bystanders.
Ng et al. (2020) [23]	To describe the sensemaking process of bystanders leading to four behavioral response types, inspired by Paull et al. (2012) [16].	Sensemaking (Weick et al., 2005) [39] and moral disengagement (Bandura et al., 1996) [26]	The model contributes to the existing literature by providing a dynamic perspective on bystander behavior. Prediction of conditions under behavioral responses are enacted, accounting for moral disengagement mechanisms. Accounting for change in bystander behaviors over time. Accounting for social contextual factors. Accounting for bystanders’ efficacy.	The authors overlooked emotions in their model. The authors overlooked the possible implication of bystanding on future events beyond the repeated bully-perpetrator interaction, namely hypervigilance of the bystander in future unrelated events and different social contexts. The model does not incorporate risk and health risk behaviors of bystanders.
Paull et al. (2012) [16]	To create a typology of bystander roles.	No framework was suggested–based on qualitative data from two separate studies.	The model contributes to the existing literature by providing a framework of bystanders’ reactions. The model is based on empirical data while other models are theoretical. The model is focused on adolescents while other models focused on employees.	The authors overlooked emotions in their model. The authors overlooked the possible implication of bystanding on future events beyond the repeated bully-perpetrator interaction. The model does not incorporate risk and health risk behaviors of bystanders The model does not have predictive mechanisms–it provides a typology.

## Data Availability

Not applicable.
